# Supplementary material for: Photoelectrochemical water splitting cells at elevated pressure using BiVO4 and platinized III-V semiconductor photoelectrodes
Source: Nat Commun. 2025 Dec 13;16:11139. doi: 10.1038/s41467-025-67294-3 (PMC12706061; doi:10.1038/s41467-025-67294-3)
Supplement: Supplementary file 1 — Supplementary Information [file 41467_2025_67294_MOESM1_ESM.pdf]

Supplementary information

Photoelectrochemical water splitting cells at elevated pressure using $BiVO_4$ and platinized III-V semiconductor photoelectrodes

Feng Liang,[1,2*] Heejung Kong,[1,3] Diwakar Suresh Babu,[1,3] Roel van de Krol,[1,3*] and Fatwa F. Abdi[1,4*]

[1] Institute for Solar Fuels, Helmholtz-Zentrum Berlin für Materialien und Energie GmbH, Hahn-Meitner-Platz 1, 14109 Berlin, Germany

[2] School of Mechanical Engineering, Xi’an Jiaotong University, No.28 Xianning West Road, Xi'an, Shaanxi 710049, China

[3] Institute for Chemistry, Technische Universität Berlin, Straße des 17. Juni 124, 10623 Berlin, Germany

[4] School of Energy and Environment, City University of Hong Kong, 83 Tat Chee Avenue, Kowloon, Hong Kong SAR, China

Correspondence and requests for materials should be addressed to Fatwa F. Abdi, Roel van de Krol and Feng Liang (emails: ffabdi@cityu.edu.hk; roel.vandekrol@helmholtz-berlin.de; feng.liang@xjtu.edu.cn)

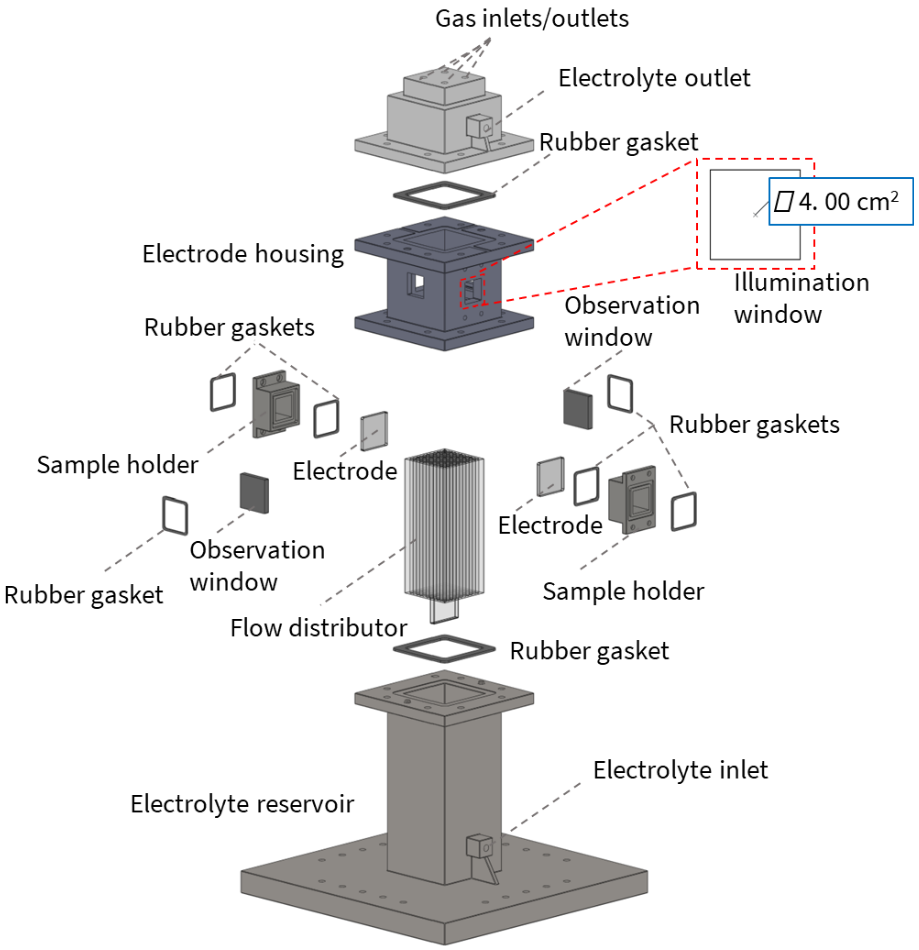

Figure S1. Exploded view of the high-pressure flow cell (HPFC) developed for this study. The electrolyte (not shown) is circulated using a rotary pump, establishing laminar flow between the electrodes via an optimized flow distributor (see Fig. S2c). The system is pressurized with external gas (N_2 or O_2, depending on the reaction), and the operating pressure is regulated by a back-pressure controller. Simulated AM1.5G illumination—optionally concentrated using Fresnel lenses—enables solar intensities up to ~10 suns. The area of the illumination window is ~4 cm^2, which determines the maximum effective area of the photoelectrodes. Observation windows are fabricated from 5 mm-thick toughened glass, allowing direct visualization of gas bubble evolution during operation at elevated pressure up to 8 bar. Standard fittings (e.g., nuts, connectors, PTFE tubing) are omitted in the schematic for clarity.

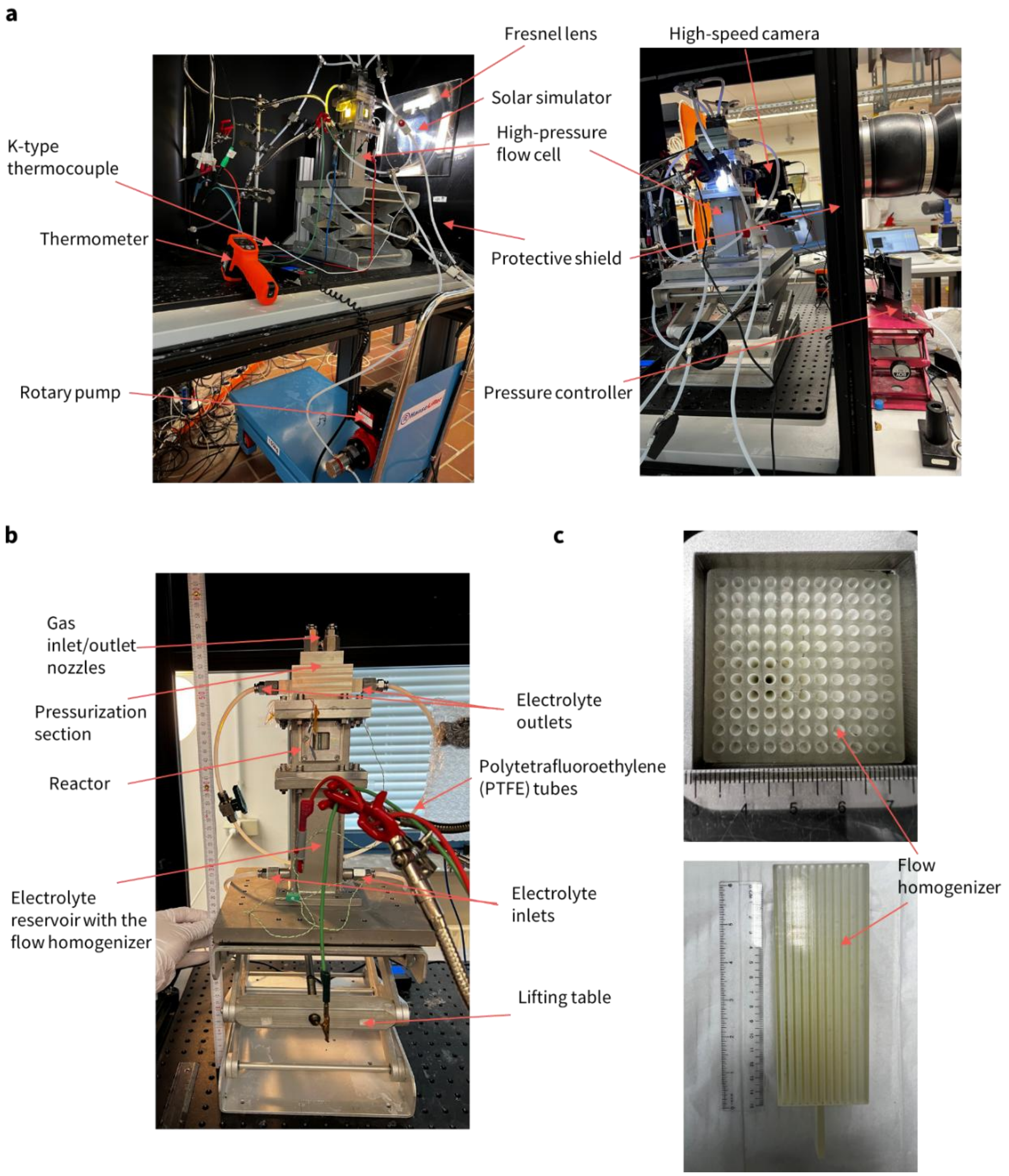

Figure S2. (a) Digital photographs of the high-pressure photoelectrochemical water splitting cell setup, (b) close-up digital photograph of the flow cell, (c) photographs of the flow homogenizer (top: cross-sectional view; bottom: side view). Detailed cell design and configurations are described in the Methods (Manuscript). In brief, the electrolyte was circulated using a rotary pump, with the electrolyte reservoir located at the bottom. An optimized flow homogenizer (or flow

distributor, panel (c)) ensured a laminar flow field in the reactor. Compressed N_2 (or O_2 in certain cases) gas was introduced to the system from the top for pressurization, with system pressure controlled via a back-pressure controller. An AM1.5G solar simulator was used and combined with Fresnel lenses of varying focal lengths to achieve a maximum solar concentration of ~10 suns. A K-type thermocouple monitored the temperature at the middle of the two electrodes. Polytetrafluoroethylene (PTFE) tubes (outer diameter: 6 mm, inner diameter: 3.5 mm, max working pressure: 14 bar) were used for liquid and gas flows. The system safely sustained pressures up to 8 bar without leaks or structural failures, and higher pressure was not tested to comply with the current laboratory safety guidelines. A protective shield was installed to safeguard personnel from any potential hazards.

Supplementary Note 1 – Solar irradiance calibration

The air mass 1.5 G (AM1.5G), 100 mW cm^{-2} simulated illumination was used as the 1 sun irradiance in our experiments. Two Fresnel lenses with focal lengths of 600 mm and 300 mm were used to concentrate solar irradiance to approximately 3 suns and 10 suns, respectively. It is important to note that our primary research focus is on the effect of pressure elevation rather than achieving a homogeneous, concentrated solar irradiance on the photoelectrode. The experimental setup with the concentrated solar irradiance is schematically shown in Fig. 1b. To calibrate the concentrated solar irradiance, we utilized a UV-vis calibrated spectrometer (USB-2000+, Ocean Optics), and the results are summarized in Fig. S3. A schematic illustration of the solar irradiance calibration is shown in Fig. S3a. Neutral density filters (ND = 1 for Fig. S3b and S3c, ND = 0.2 for Fig. S3d) were used to prevent saturation during spectral measurement. Fig. S3b presents the measured irradiance at different spots (x = distance from the center of the light) when different Fresnel lenses are used, while Fig. S3c shows the average irradiance over a ~20 mm length scale, corresponding to the size of our photoelectrode. Our measurements indicate that the 300 mm focal length Fresnel lens achieves approximately 10 suns, whereas the 600 mm lens provides ~3 suns of solar irradiance. The spectra of the simulated sunlight are shown in Fig. S3d, obtained using a neutral density filter with ND = 0.2. The blue spectrum corresponds to measurements taken without a Fresnel lens, while the red and dark gray spectra represent measurements with the 600 mm and 300 mm Fresnel lenses, respectively. Other than the intensity (i.e., counts), the spectra remain similar. This confirms that the Fresnel lenses effectively increase solar concentration without altering the spectral profile, making them suitable for investigating the effects of pressure on the photoelectrode under elevated solar irradiance.

The simulated solar irradiance with the Fresnel lenses varied depending on the cell position (see Fig. S3a), particularly when using the 300 mm Fresnel lens (as shown in Fig. S3b and S3c). To minimize the impact of cell positioning at higher solar concentrations, additional alignment measures were implemented. Specifically, three aligners were designed to precisely position the Fresnel lens (see Fig. S4a), and similar alignment techniques were applied to the flow cell. The resulting illuminated area under ~10 suns is shown in Fig. S4b, covering approximately 40 cm^2—about ten times the size of our photoelectrode (~4 cm^2). Additionally, to account for potential variations in cell positioning during different tests, the photocurrent values reported in Fig. 3b, Fig. 4a, and Fig. 4c (Manuscript) were normalized against the 1 sun measurement taken at the same location. This normalization ensures consistency and eliminates positional discrepancies across experiments.

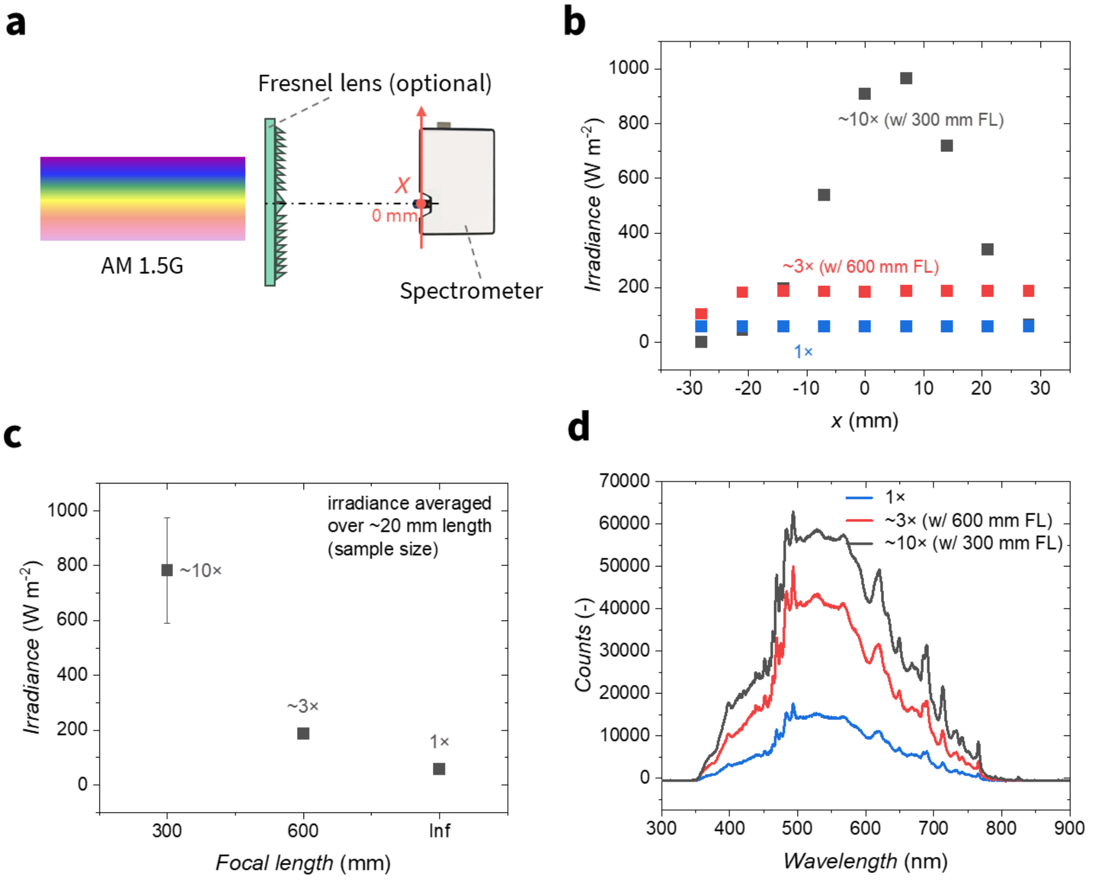

Figure S3. (a) Schematic illustration of the solar irradiance calibration. (b) Measured solar irradiance under different Fresnel lens configurations. A neutral density filter (ND = 1) was used for all three solar concentrations. (c) Averaged solar irradiance over a ~20 mm length scale, corresponding to the size of the $BiVO_4$ photoelectrode, with ND = 1 included during measurement. (d) Spectra of the solar simulator under different Fresnel lens and neutral density filter (ND = 0.2) configurations. AM1.5G was used to simulate 1 sun irradiation. The measurements were conducted at room temperature (~25 °C).

a b

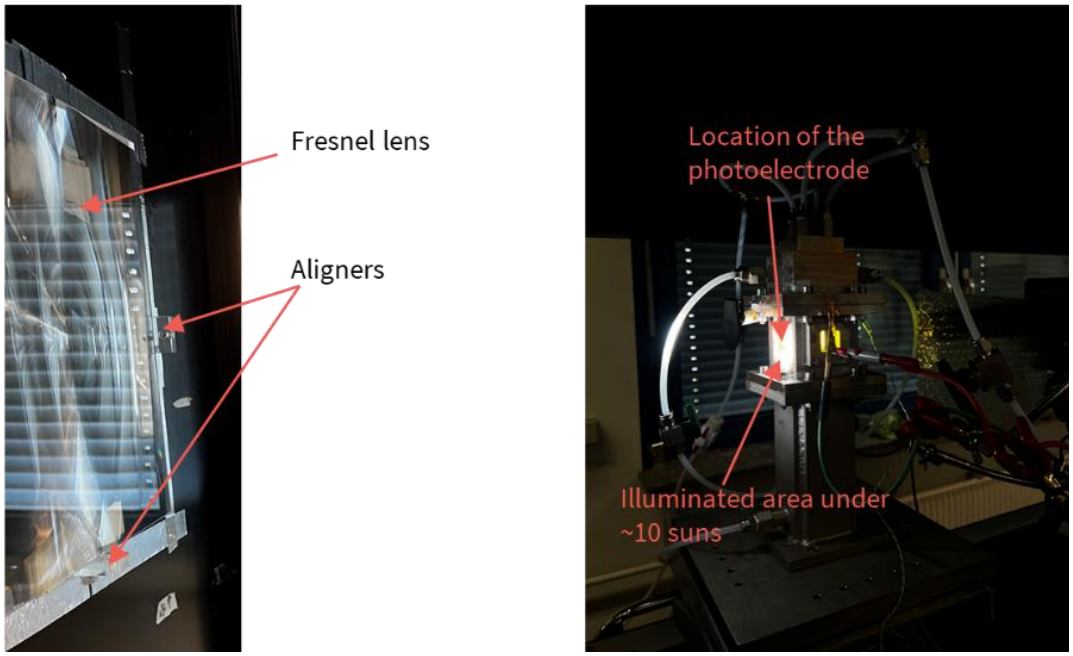

Figure S4. (a) Photograph of the Fresnel lens and aligners. (b) The HPFC under ~10 suns illumination using a Fresnel lens with a ~300 mm focal length, which produces the smallest illumination area in our experiments (~40 cm²). This illuminated area is approximately 10 times larger than the $BiVO_4$ photoelectrode (~4 cm²) used in our measurements.

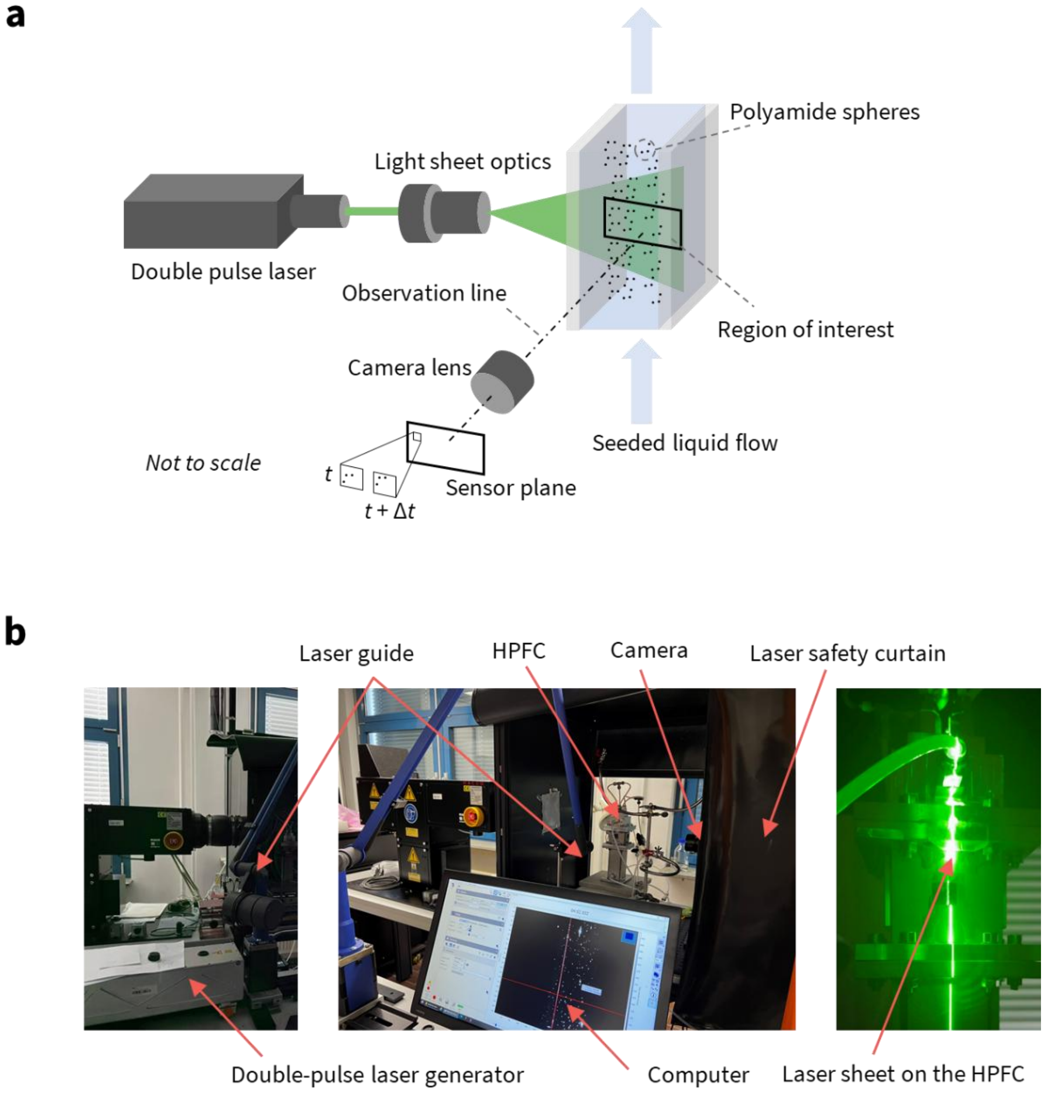

Figure S5. (a) Schematic illustration of the particle image velocimetry (PIV) measurement, (b) digital photograph of the experiment setup. The photo on the right shows the laser sheet illuminating the flow cell, with the camera positioned on the right side of the flow cell to observe particle movement from a perpendicular angle. The software running on the computer displays a "live view" of the measurement, with the white dots indicating the captured particles under illumination. The PIV measurement was conducted inside a protective shield and laser safety curtain, with laser safety goggles worn by personnel during the experiments. The measurements were conducted at room temperature (~25 °C).

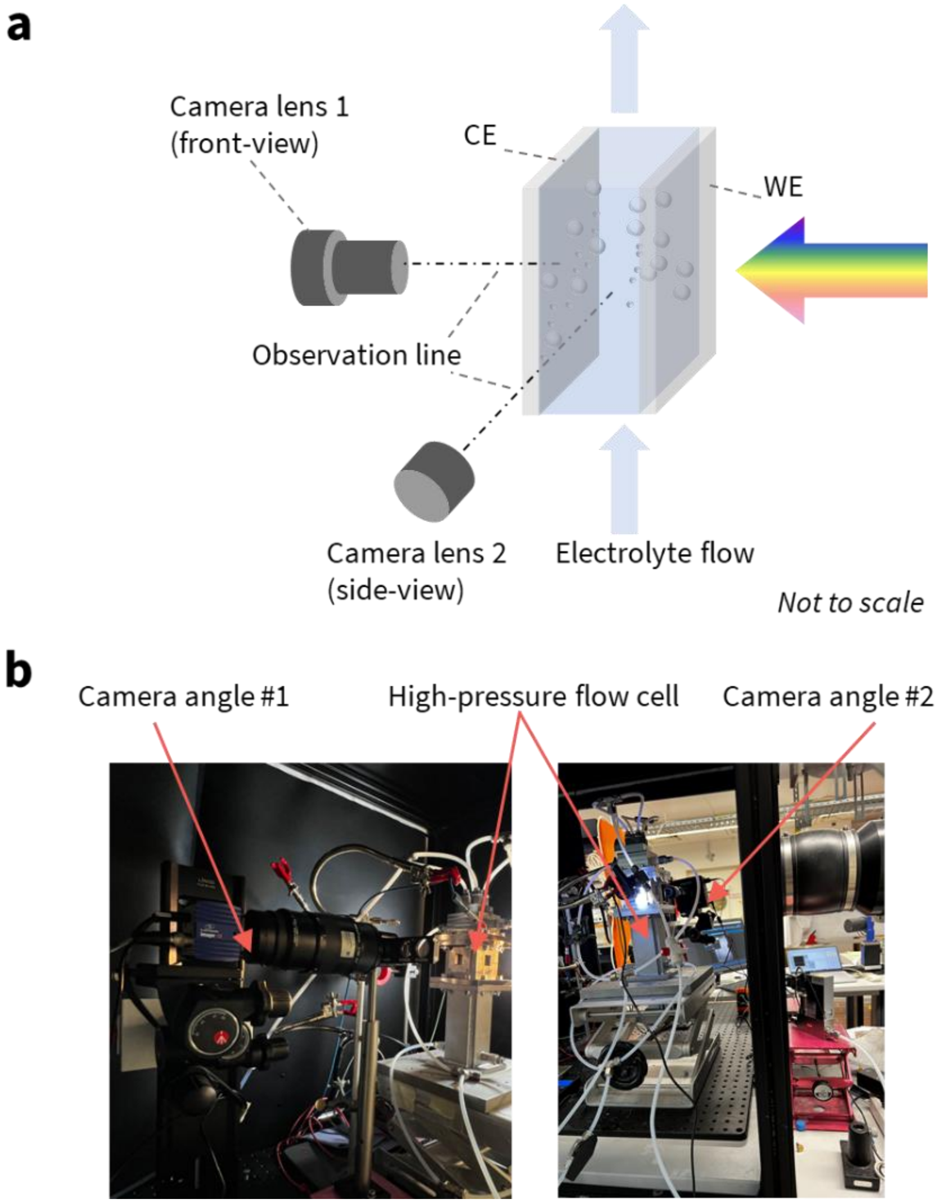

Figure S6. (a) Schematic illustration of the multi-angle bubble imaging setup. (b) Photographs of the bubble imaging setup. The detailed introduction of the gas bubble visualization is described in the Methods (Manuscript). Briefly, two cameras were used to capture gas bubble evolution from both side and front (or back) views. To address prevent saturation (especially at higher concentrations), neutral density filters with varying ND factors were placed between the camera lens and the object (not visible in the photographs).

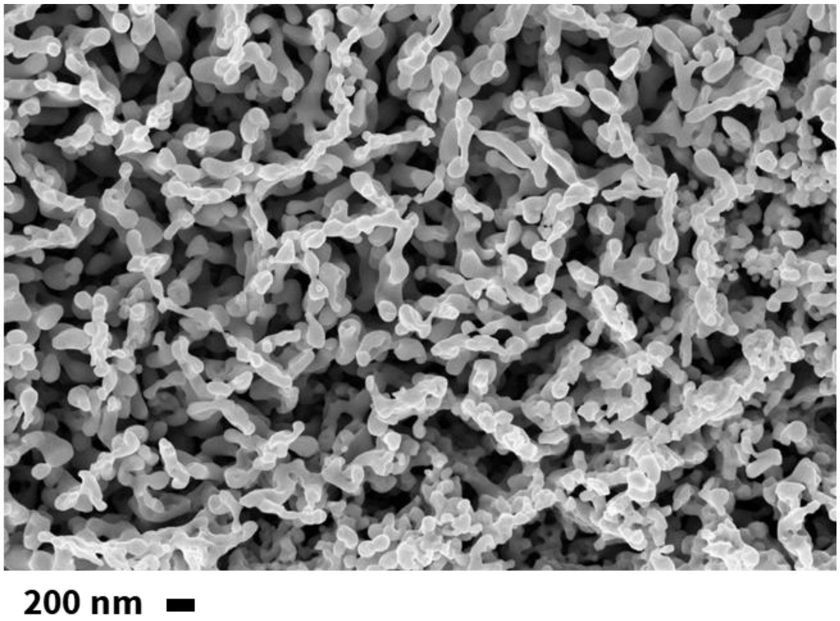

Figure S7. Scanning electron microscopy (SEM) image of the electrodeposited nanoporous $BiVO_4$ film.

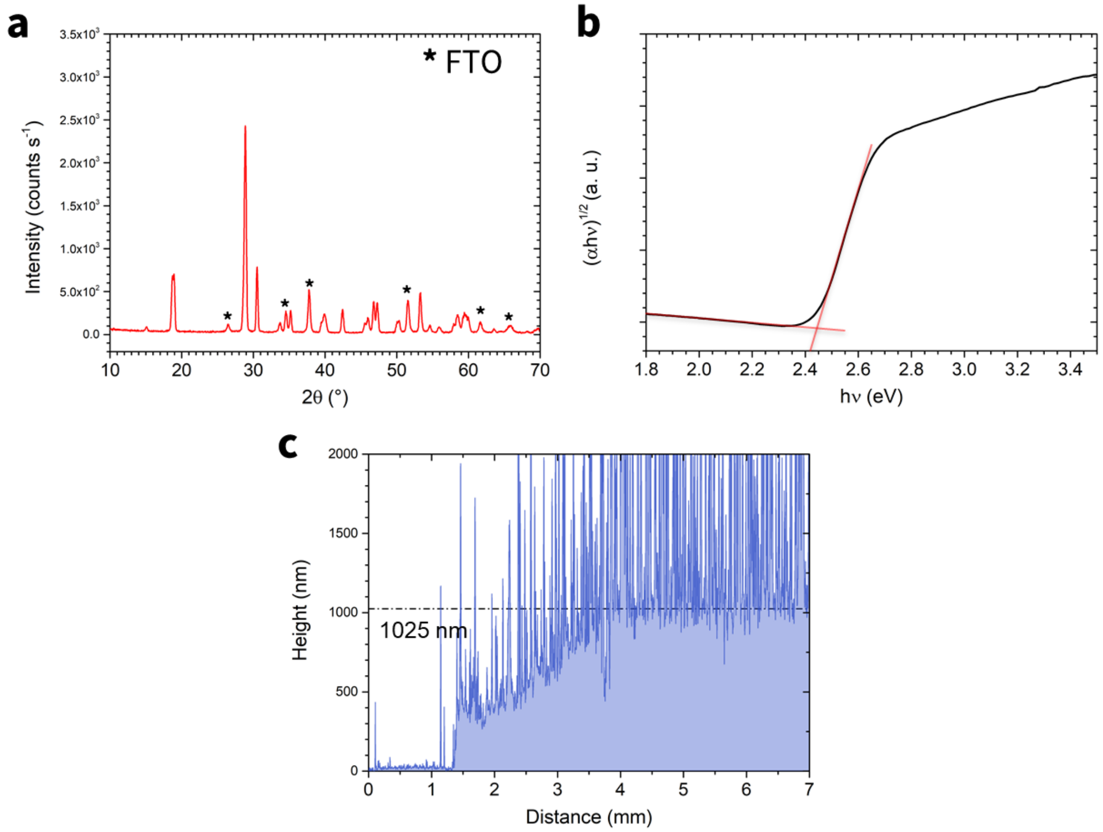

Figure S8. (a) XRD pattern of the $BiVO_4$ film and the FTO substrate. (b) Tauc plot of the $BiVO_4$ film for the indirect bandgap estimation. (c) film profiling measurement (DEKTAK 8 profilometer, Veeco). X-ray diffraction (XRD) was conducted using an X-ray diffractometer (X'Pert, PANalytical). A Cu Kα radiation with a wavelength of 1.5406 Å was employed, and the incident angle of the X-ray was set to 2°.

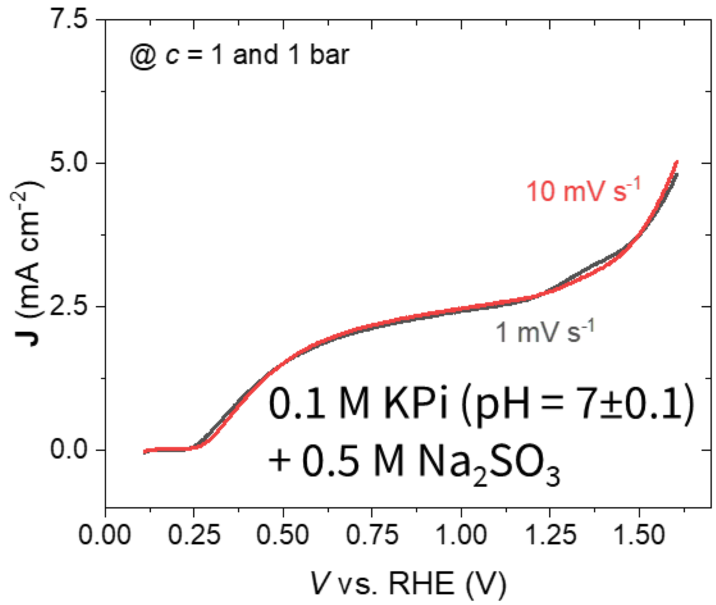

Figure S9. Comparison of the different LSV scan rate (1 vs. 10 mV s^{-1}) with the hole scavenger. AM1.5G solar irradiance was applied and the cell pressure was maintained at 1 bar with the electrolyte purged using O_2. A back-illuminated $BiVO_4$ photoelectrode was used for the measurement. The electrolyte was circulated using a rotary pump at a constant flow rate of approximately 4.6 mL s^{-1}. The measurements were conducted at room temperature (~25 °C). No iR corrections was done for the reported voltammogram.

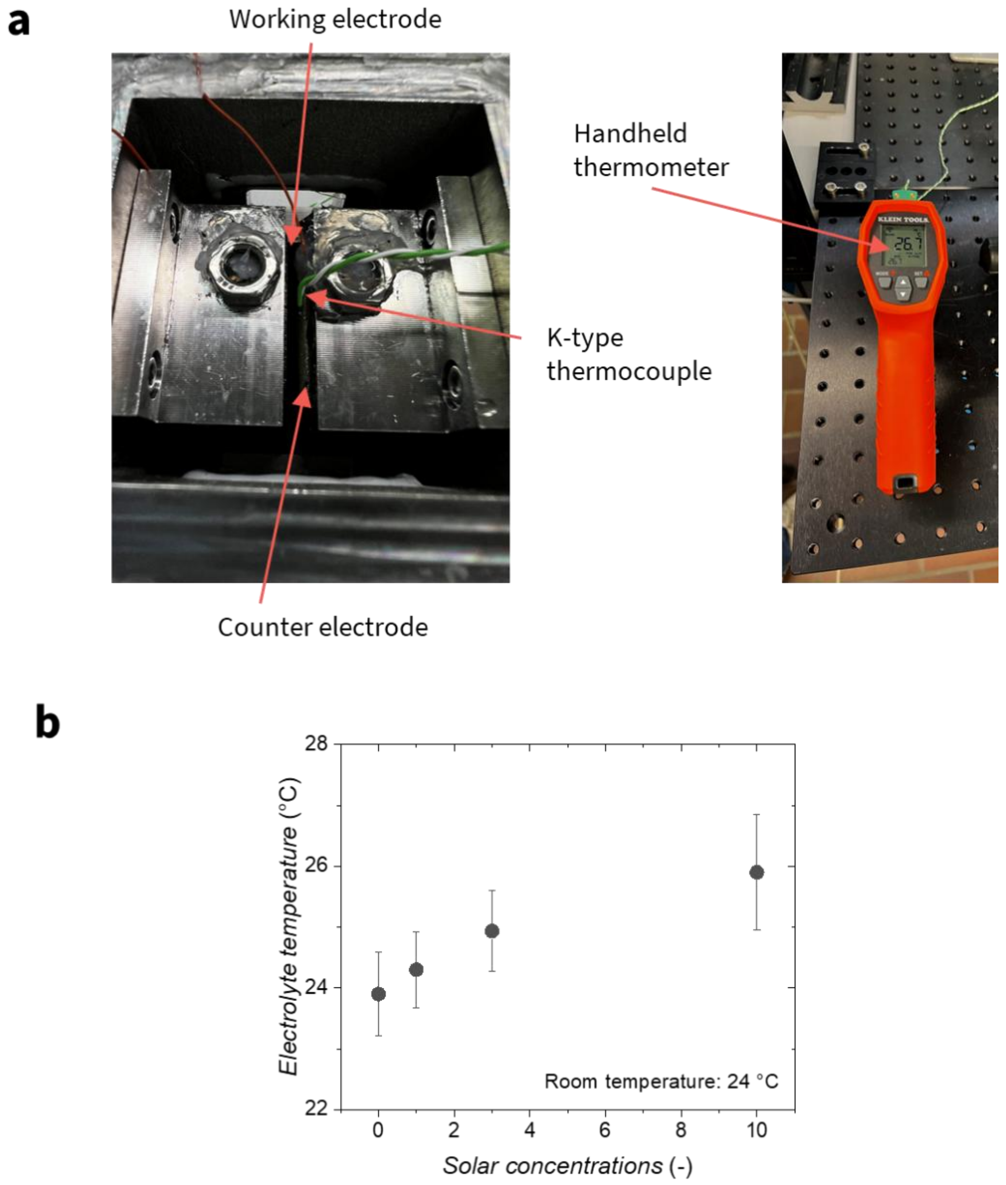

Figure S10. (a) Digital photographs of the temperature measurement setup. (b) Electrolyte temperature measured during the experiments at various solar concentrations, c. c = 0 refers to the dark condition, while c = 1 denotes AM 1.5G, and higher concentrations follow accordingly. Note that the temperature results in panel (b) were measured during LSV experiments, with a ~5-minute break in between experiments to prevent continuous heating of the electrolyte. The K-type thermocouple was positioned at the exit of the electrolyte flow, midway between the two electrodes.

Temperature values were recorded using a handheld thermometer. Error bars represent the standard deviation from three independent measurements. The measurements were conducted at room temperature (~24 ℃).

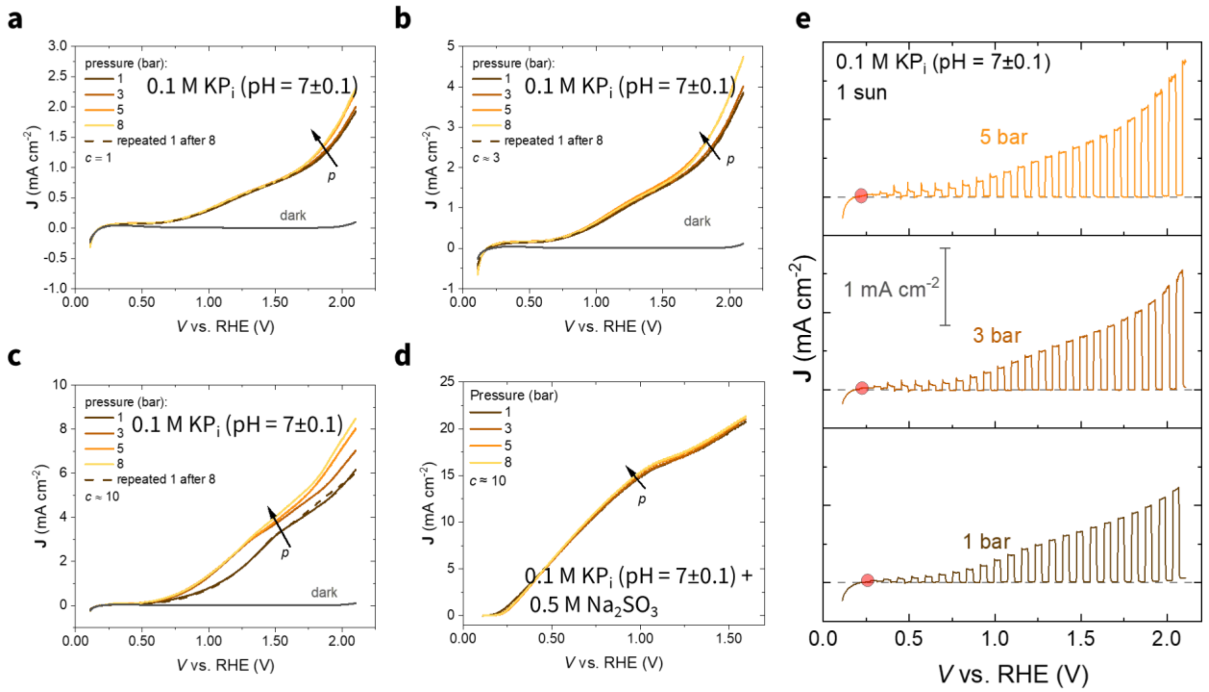

Figure S11. (a) LSV scans of the back-illuminated $BiVO_4$ photoelectrode at different pressure under AM1.5G (c = 1), the electrolyte was a 0.1 M KP_i (pH = 7±0.1) solution. (b) and (c) show the same results as in panel (a) but under concentration of $c \approx 3$ and $c \approx 10$, respectively. Additional measurement at 1 bar (dashed curve) was performed after that at 8 bar to check for reproducibility. (d) Same as (a) but with the addition of the hole scavenger (0.5 M Na_2SO_3). (e) Chopped-light (1 sun) LSVs of $BiVO_4$ photoelectrode at different pressures. The onset potentials for each curve are indicated with the red circles. The compressed O_2 gas was used for the electrolyte purging and cell pressurization. The scan rate for the LSV was 20 mV s^{-1} and the electrolyte was circulated by a rotary pump at a constant flow rate of approximately 4.6 mL s^{-1}. The measurements were conducted at room temperature (~25 °C). No iR corrections was done for the reported voltammograms.

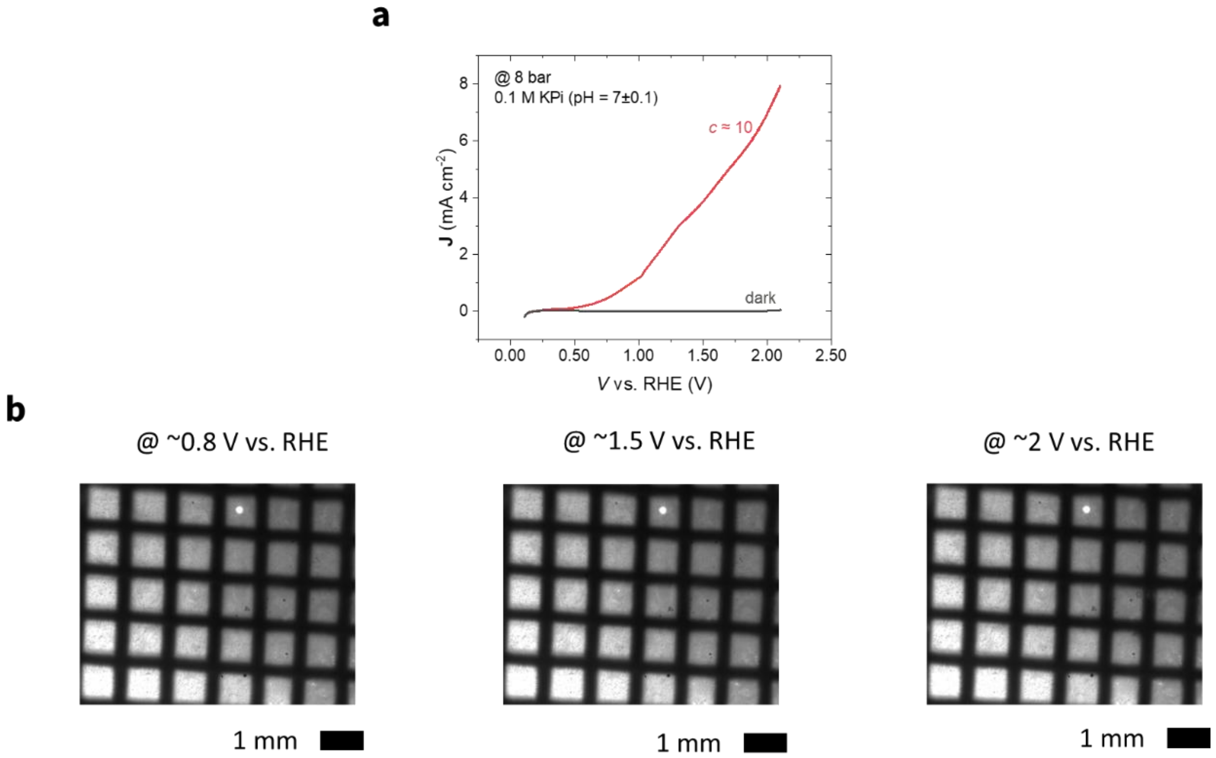

Figure S12. (a) LSVs at 8 bar under dark vs. $c \approx 10$ solar concentration. (b) Representative images of the "bubble-free" electrodes taken at different potentials during LSV measurement under ~10 suns. The $BiVO_4$ photoelectrode (working electrode) appears as the grey background in the images, while the counter electrode (Pt mesh) is visible as the black grid. The electrolyte was purged and pressurized with compressed oxygen. The electrolyte was 0.1 M KP_i (pH = 7±0.1), and the flow rate was kept at ~4.6 mL s^{-1}. The scale bars in the images represent 1 mm. The measurements were conducted at room temperature (~25 °C). No iR corrections was done for the reported voltammogram.

Supplementary Note 2 – Laplace pressure of gas bubbles with different sizes

The Laplace pressure is the pressure difference between the inside and the outside of a curved surface that forms the boundary between two fluid regions.[1] In our case, this pressure difference is caused by the surface tension of the interface between liquid and gas. Here we assume that the gas bubble/electrolyte interface is spherical, and the Laplace pressure of the interface determined from the Young-Laplace equation can be written as:[2]

$$\Delta p = p_{\text{inside}} - p_{\text{outside}} = p_{\text{bub}} - p_{\text{hydro}} = \gamma \frac{2}{R_{\text{bub}}} \quad \text{(S1)}$$

where p_{bub} is the gas pressure inside the bubble, p_{hydro} denotes the hydrostatic pressure of the surrounding electrolyte, R_{bub} is the radius of the spherical bubble interface, and γ is the surface tension coefficient (assume here as 72 mN m^{-1}).[3]

Fig. S13 presents the calculated Laplace pressure between the internal and external of the gas bubble interface as a function of bubble radius. The Laplace pressure increases as the bubble size decreases, indicating that the pressure inside the gas bubbles is higher when bubbles are smaller. For gas bubbles with a radius of 0.1 µm, the Laplace pressure is ~20 bar. Since the pressure range during our PEC measurements is between 1 and 8 bar, this pressure variation in the PEC cell is unlikely to cause a significant effect on the micro/nano-bubbles.

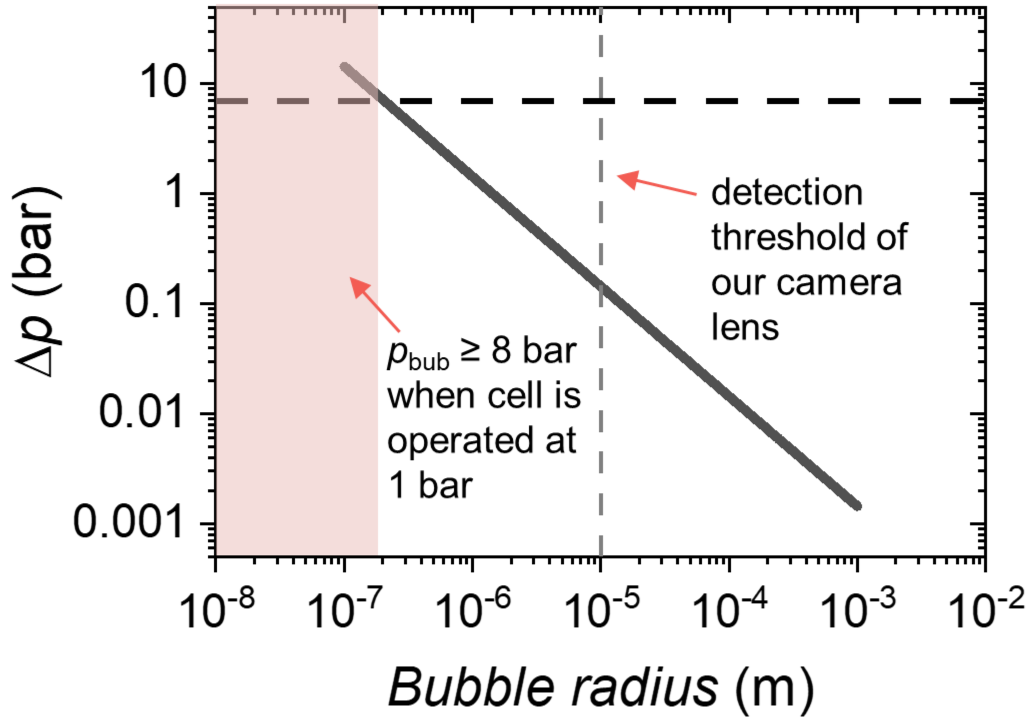

Figure S13. Laplace pressure of the gas bubble interface as function of bubble size. The red-shaded part represents the bubble radii smaller than ~0.2 µm, for which the Laplace pressure exceeds 7 bar (horizontal dashed line). This indicates that the internal pressure of the gas bubble, p_{bub}, is higher than 8 bar when the cell operates at 1 bar. The bubble interface was assumed to be spherical with the surface tension coefficient set at 72 mN m^{-1}.[3] The detection threshold of the bubble radius with our microscopic camera lens is about 10 µm (vertical dashed line).

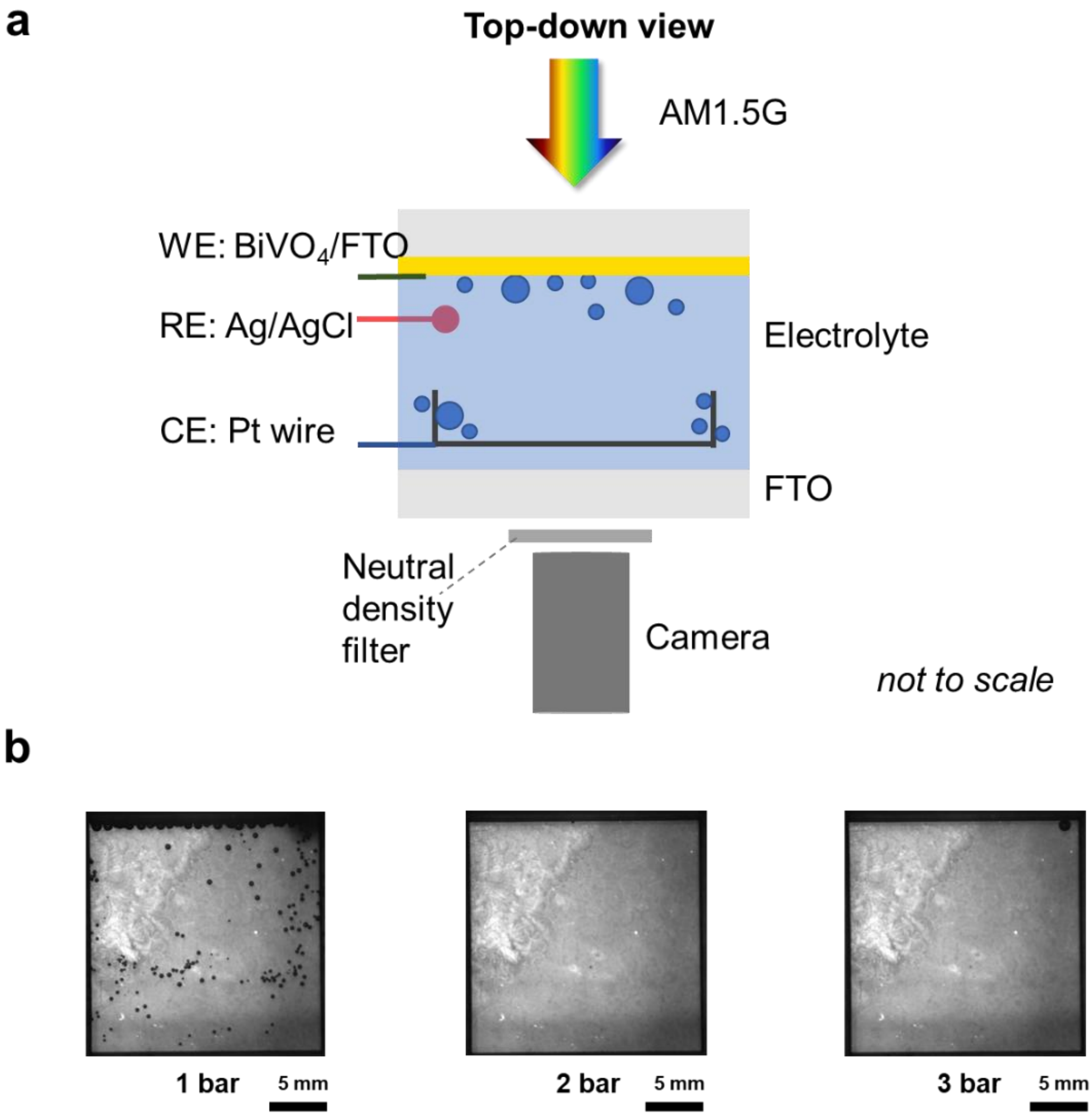

Figure S14. Macroscopic shadowgraphy of oxygen bubble evolution from the $BiVO_4$ photoanode. (a) Schematic of the measurement setup. (b) Representative images of bubble formation at 1, 2, and 3 bar. The working electrode ($BiVO_4$, 2 cm × 2 cm active area) is identical to that used in the main study. A platinum wire was employed as the counter electrode to minimize visual obstruction, and a saturated Ag/AgCl electrode served as the reference. The PEC cell was operated galvanostatically at 0.5 mA cm^{-2} in 0.1 M potassium phosphate buffer (KP_i, pH = 7±0.1), with the electrolyte held static. Imaging was conducted using a Zeiss Makro-Planar T* 2/100 lens coupled

to a high-speed camera (HS5-Q, 2560 × 2048 pixels), yielding a bubble detection threshold of approximately 45–50 μm. Note that gas bubbles observed at the top of the frames correspond to the region of electrical contact, where a small amount of silicone epoxy was applied for insulation. The scale bars represent 5 mm. The measurements were conducted at room temperature (~25 °C).

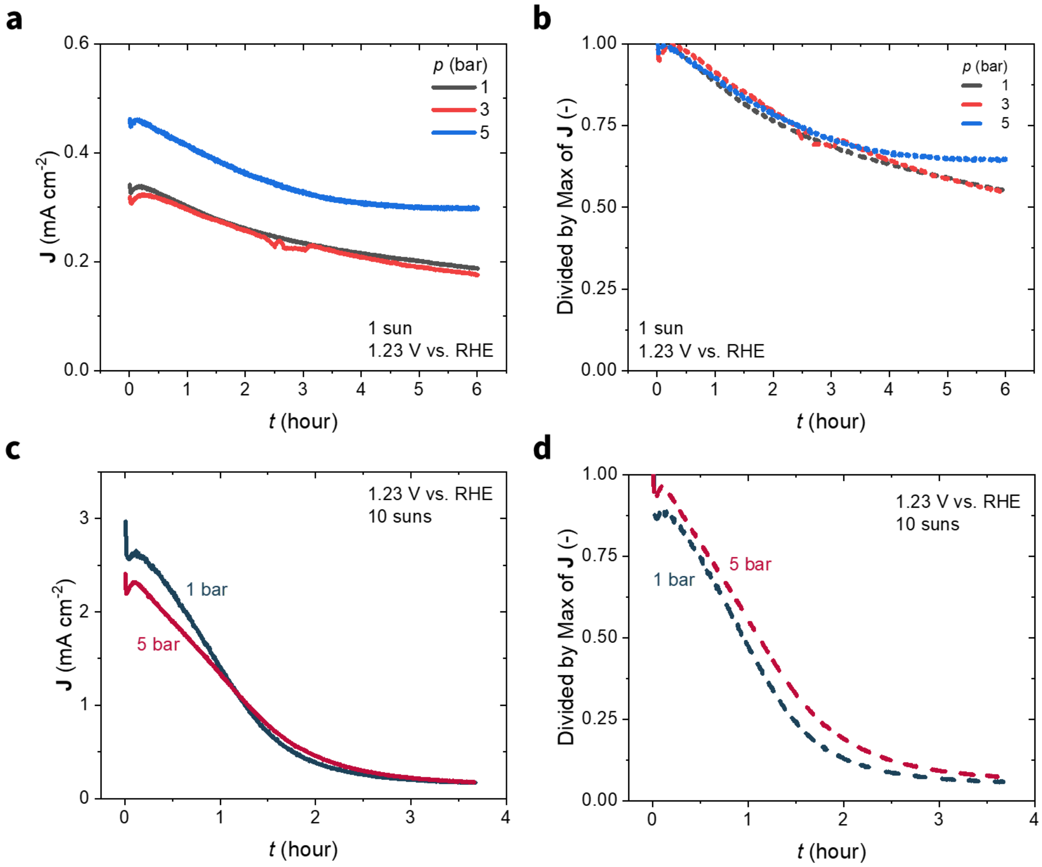

Figure S15. Stability measurements of the back-illuminated, $BiVO_4$-based PEC water splitting cell under different pressures. (a) Chronoamperometry (CA) measurements under AM1.5G 1 sun illumination; (b) the same CA data divided by the respective $\mathbf{J}_{max}$ for clearer comparison. (c) and (d) present the corresponding CA results under concentrated illumination (10 suns). In all tests, undoped and uncatalyzed $BiVO_4$ was used as the photoanode, with a platinum mesh as the counter electrode (CE), and Ag/AgCl as the reference electrode. The PEC cells were operated potentiostatically at 1.23 V vs. RHE in a 0.1 M potassium phosphate (KP_i) buffer solution (pH = 7±0.1), with an electrolyte flow rate of 4.6 mL s^{-1} was maintained during the stability measurements. The CA test was terminated upon ~50% photocurrent loss at 1 sun and near-

complete degradation at 10 suns. The measurements were conducted at room temperature (~25 °C).

Supplementary Note 3 – Thermal effect of the concentrated solar irradiance during stability tests

To evaluate the thermal effects associated with prolonged operation under concentrated sunlight, we developed a heat transfer model as described below.

Model definition and assumptions

We simplified the PEC cell geometry (see Fig. 1b, Manuscript and Fig. S1) to simulate the coupled heat transfer in solids and fluids. The $BiVO_4$/FTO photoanode was modelled as a quartz substrate with equivalent thickness. The fluid domain (electrolyte) was assumed to be liquid water under fully developed laminar flow, with inlet velocity taken from PIV measurements (Fig. 2a, Manuscript). An inward heat flux of 5600 W m^{-2} (corresponding to 56% absorption under 10 suns, per Ref. [4]) was applied to the illuminated surface. The edges of the electrode, which are in contact with the rubber gasket and stainless-steel cell body in our setup (see Fig. S1), as well as the right-side boundary of the fluid domain (in contact with the counter electrode), were treated as adiabatic boundaries, as shown in Fig. S16a and S16c. As a result, the simulated surface temperature of the photoelectrode represents a worst-case scenario for thermal accumulation.

Fluid dynamics

The following continuity equation for incompressible Newtonian fluids that conserves mass was considered,

$$\rho \nabla \cdot \mathbf{v} = 0 \tag{S2}$$

where $\mathbf{v}$ is the velocity field. The density of the fluid phase (ρ) is constant. This means that the only way to change the mass of the computational domain (Ω) is by convection, which is expressed in the following equation.

$$\rho(\mathbf{v} \cdot \nabla)\mathbf{v} = \nabla \cdot [-p\mathbf{I} + \mathbf{K}] + \mathbf{F} \tag{S3}$$

$$\mathbf{K} = \mu(\nabla\mathbf{v} + (\nabla\mathbf{v})^{\mathrm{T}}) \quad \text{(S4)}$$

Momentum conservation was solved using the Navier-Stokes equation in (eqn. S3). p is pressure, **I** is the identify tensor, while **F** is the external volume forces, e.g., gravity. **K** is the viscous stress tensor, and expressed in (eqn. S4), while μ is dynamics viscosity.

Boundary conditions in the fluid dynamics model

The liquid inlet was considered as fully-developed laminar flow,

$$\mathbf{v} = \left(0,\ 1.5\mathbf{U}\left\{1 - \left(\frac{2x}{L_x}\right)^2\right\}\right) \quad \text{(S5)}$$

where L_x is the width of the flow channel and equals 4 mm, and **U** denotes the average inlet flow velocity. The **U** values were obtained from our PIV measurement presented in Fig. 2a (Manuscript) and set as 7.33 mm s^{-1}.

The outlet pressure was set as 0 bar and 4 bar, respectively, to mimic the pressure settings of 1 bar and 5 bar in our experiments. The side walls were treated as no slip boundaries, as shown in Fig. S16b.

Heat transfer in solids and fluids

The heat transfer in the solid part was solved using the following equation.

$$d_z\rho C_p\mathbf{v}\cdot\nabla T + \nabla\cdot\mathbf{q} = d_zQ + q_0 \quad \text{(S6)}$$

$$\mathbf{q} = -d_zk\nabla T \quad \text{(S7)}$$

Here, d_z is the thickness of the domain and equals 30 mm. C_p is the specific heat capacity of the fluid, $\mathbf{v}\cdot\nabla T$ represents the convection term, and $\nabla\cdot\mathbf{q}$ solves heat conduction (divergence of heat flux). The heat flux **q** is defined in (eqn. S7), with k representing the thermal conductivity of the material ($k = 1.4\ \mathrm{W\ (m\ K)^{-1}}$ for quartz glass). On the right-handed side of the equation, d_zQ denotes the volumetric heat source, and q_0 is the surface heat source.

For the fluid domain, the same equations were used, with nonisothermal flow assumed. The viscous dissipation term was included as

$$Q_{\mathrm{vd}} = \tau : \nabla \mathbf{v} \tag{S8}$$

where Q_{vd} is the dissipative heat generation term, τ is the viscous stress tensor.

Boundary conditions in the heat transfer model

An inward heat flux of 5600 W m^{-2} (corresponding to 56% absorption under 10 suns, per Ref. [4]) was applied at the illuminated section of the $BiVO_4$ photoanode. The edges of the electrode, which are in contact with the rubber gasket and stainless-steel cell body in our setup (see Fig. S1, *Supplementary Information*), as well as the right-side boundary of the fluid domain (in contact with the counter electrode), were treated as adiabatic boundaries, as shown in see Fig. S16c. The numerical settings for other boundaries are shown in Fig. S16c.

Numerical treatment

The model was solved in fully coupled mode using COMSOL Multiphysics® 6.2 with the PARDISO solver. A P1+P1 scheme was used for pressure-velocity coupling, and time integration was performed using the generalized alpha method with adaptive time stepping. The computational domain was discretised into 79,927 elements, yielding an average mesh quality of 0.86 (with 1.0 indicating an ideal mesh). Simulations were run on a high-performance workstation (Intel Xeon E5-2650 v2, 64 cores, 256 GB RAM) with a relative tolerance of 0.005.

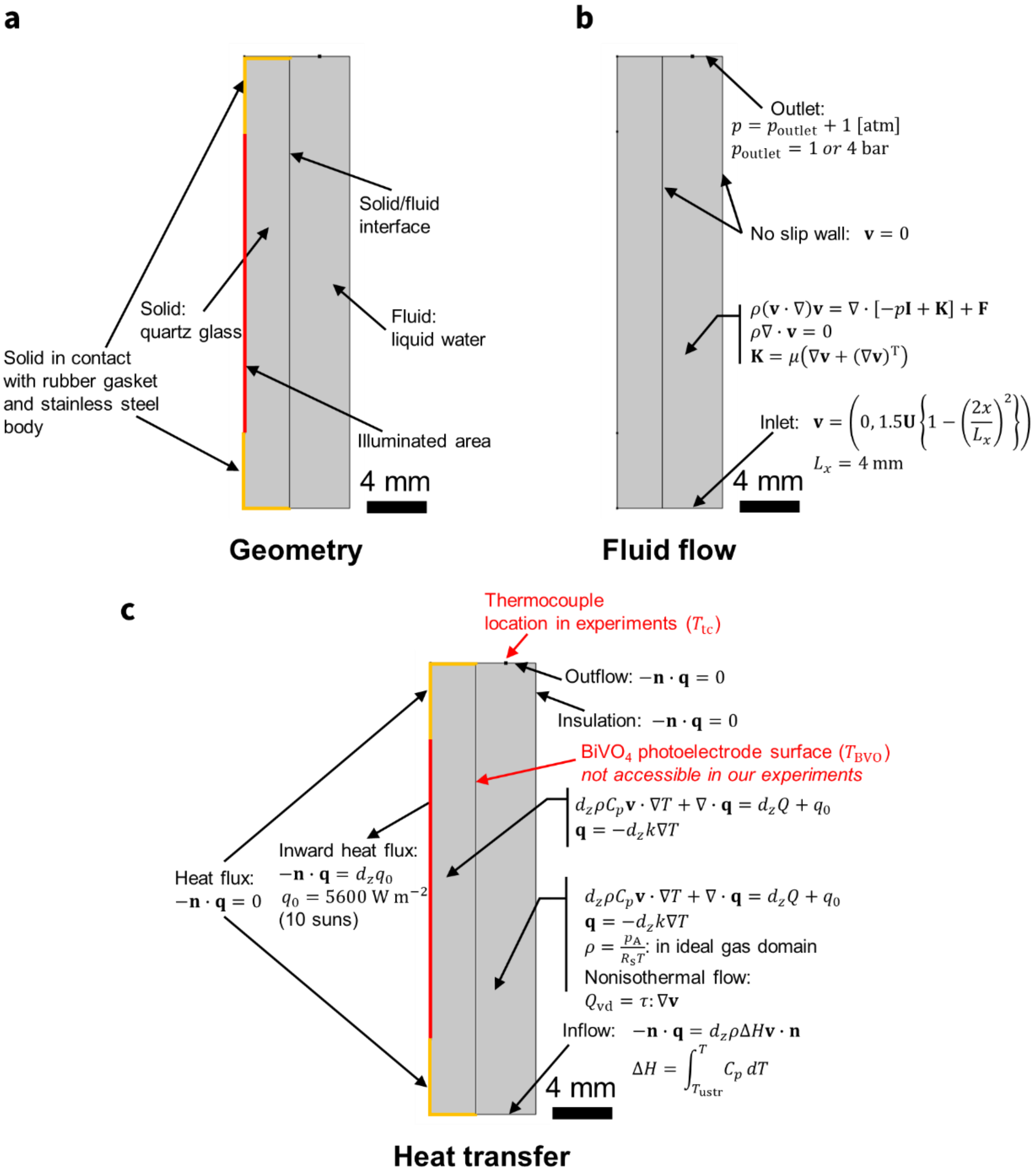

Figure S16. Two-dimensional heat transfer model used for our simulations. The simplified geometry of our setup is shown in (a). (b) Governing equations and boundary conditions for the fluid flow model. (c) Heat transfer model in the solid and fluid domains. The $BiVO_4$/FTO sample was modelled using a quartz glass piece, with the electrolyte treated as liquid water. An inward

heat flux of 5600 W m^{-2} (corresponding to 56% absorption under 10 suns, per Ref. [4]) was applied at the illuminated section of the $BiVO_4$ photoanode. The edges of the electrode, which are in contact with the rubber gasket and stainless-steel cell body in our setup (see Fig. S1), as well as the right-side boundary of the fluid domain (in contact with the counter electrode), were treated as adiabatic boundaries in panels (a) and (c). As a result, the simulated surface temperature of the photoelectrode represents a worst-case scenario for thermal accumulation. The liquid flow is considered as the fully-developed laminar flow, with the average velocity obtained from our PIV measurements (shown in Fig. 2a, Manuscript).

Figure S17a compares the simulated surface temperature with experimental bulk measurements. Both show ~5 °C rise under steady-state illumination, validating the model′s ability to capture thermal behavior. Figure S17b-S17d show a maximum surface temperature rise of ~13 °C at the photoelectrode in the end of the 3.5 hours stability tests. While this estimate may be somewhat overestimated due to simplified boundary assumptions—namely, adiabatic treatment of the electrode sealing interfaces and the electrolyte boundary adjacent to the counter electrode—it nonetheless highlights the potential for localized heating at the photoelectrode surface. Such a temperature increase can indeed facilitate reaction kinetic[5] and accelerate photoelectrode material degradation[6]. These findings emphasize the importance of thermal management in high-pressure PEC systems under concentrated sunlight. Advanced cooling strategies, such as those demonstrated in Ref. [7] and Ref. [4,8], can be implemented to prevent photoelectrode overheating under concentrated sunlight. Importantly, the similar thermal trends observed in both our measurements and simulations at different pressures support the validity of our comparative performance analysis, as illustrated in Fig. S17.

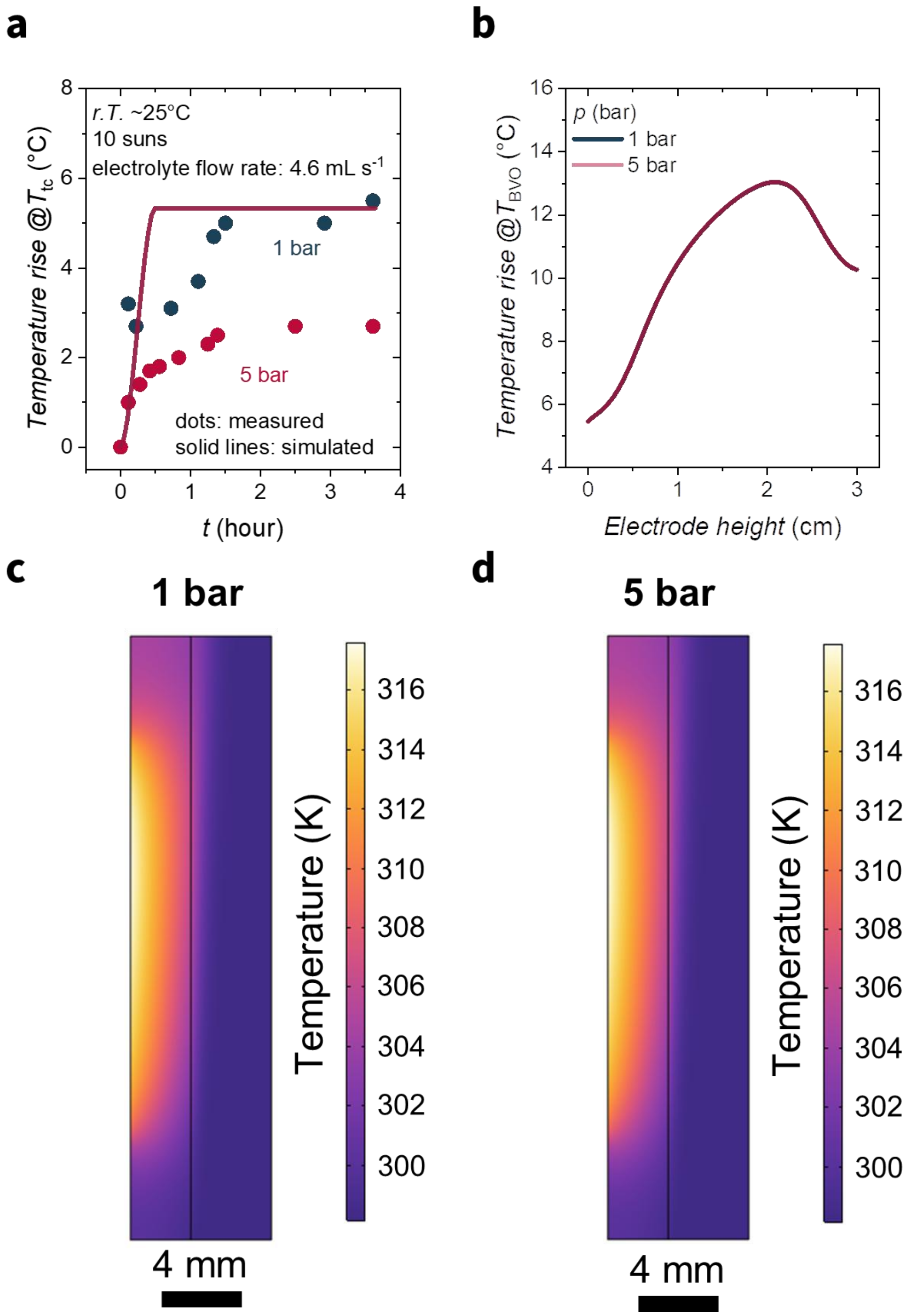

Figure S17. Thermal measurement and simulation results during the stability test under ~10 suns illumination. (a) Comparison between simulated and measured temperature rise in the bulk

electrolyte at different pressures. (b) Simulated surface temperature of the photoelectrode after ~3.5 hours stability tests. Temperature distribution colormaps at the end of the test at (c) 1 bar and (d) 5 bar. During the stability measurement, undoped and uncatalyzed $BiVO_4$ was used as the photoanode, with a platinum mesh as the counter electrode (CE), and Ag/AgCl as the reference electrode. The PEC cells were operated potentiostatically at 1.23 V vs. RHE in a 0.1 M potassium phosphate (KP_i) buffer solution (pH = 7±0.1), and an electrolyte flow rate of 4.6 mL s^{-1} was maintained during the stability measurements. A K-type thermocouple was used to measure the electrolyte temperature, according to the digital photographs of the measurement setup in Fig. S10a.

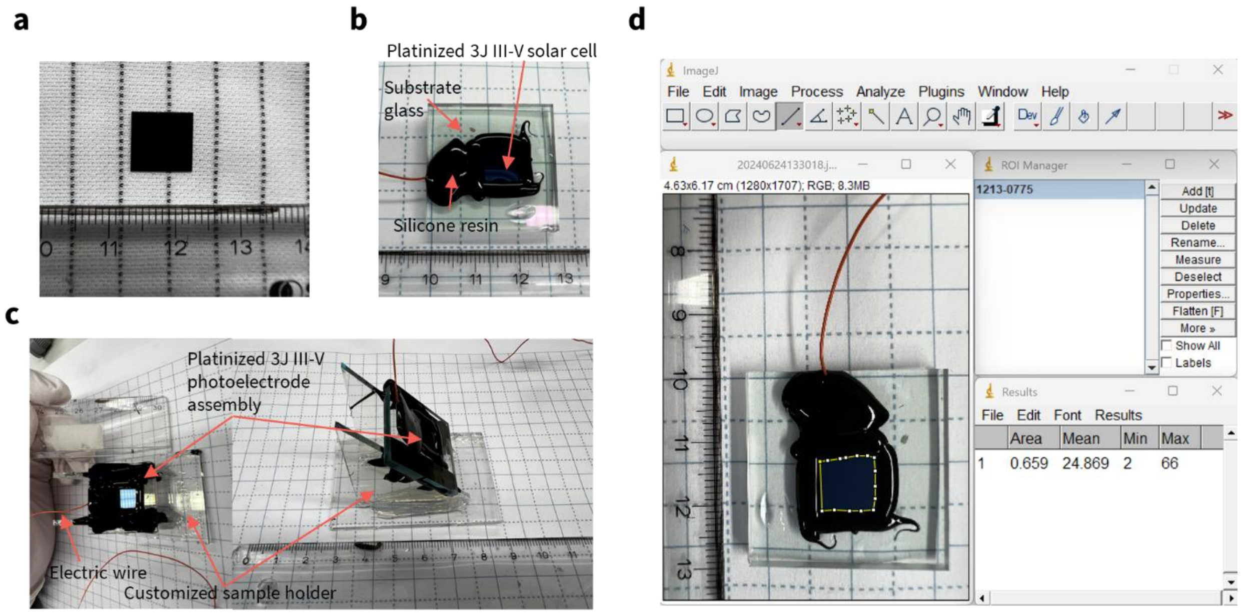

Figure S18. (a) Platinized triple-junction (3J) III-V PEC cell. (b) Photoelectrode assembly. (c) Photoelectrode assembly with the customized sample holder. A detailed description of the sample preparation procedure is provided in the Methods (Manuscript). Briefly, electrical contact was made using Ag tape, conductive epoxy, and a copper wire, with all connections sealed properly with silicone resin (as indicated in panel (b)). The resulting photoactive area can be observed by the reflected area in (c), which was approximately 0.6 – 0.7 cm^2, measured using ImageJ with details shown in (d). A customized sample holder was used to enable 45° illumination of the photoelectrode assembly. Both the photoelectrode assembly and the sample holder were sealed to the side wall of the reactor using silicone resin (see Fig. S1). No liquid leakage or glass cracks occurred within our pressure range (1 – 8 bar).

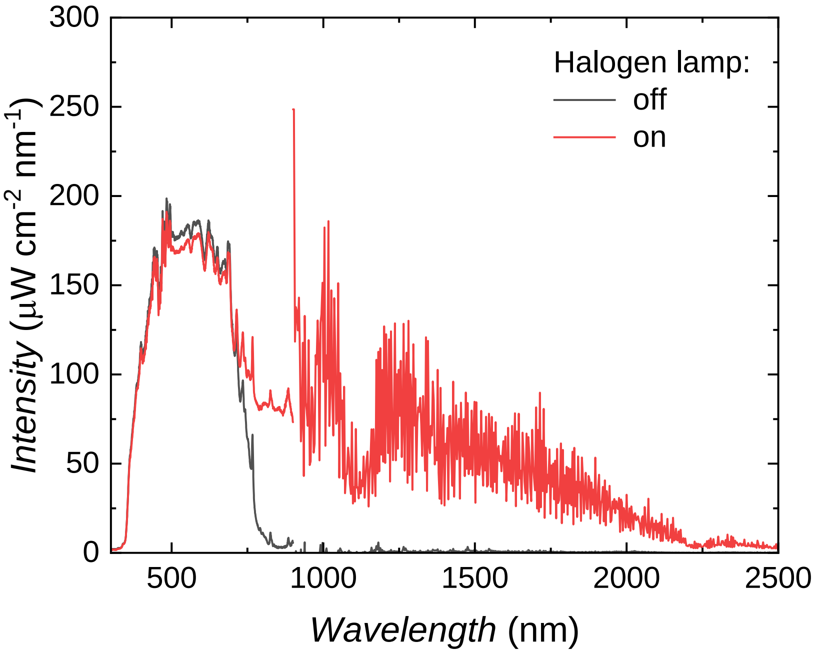

Figure S19. Spectra of the solar simulator with and without the halogen lamp. The 3J III-V solar cell from AZUR SPACE Solar Power GmbH consists of GaInP/GaAs/Ge junctions with bandgaps of 1.8 eV, 1.4 eV, and 0.7 eV, respectively, enabling absorption across the entire spectrum, including the infrared region.[9] Our dual light source solar simulator is equipped with an optional halogen lamp to provide the infrared spectrum, as shown in the measurement above.

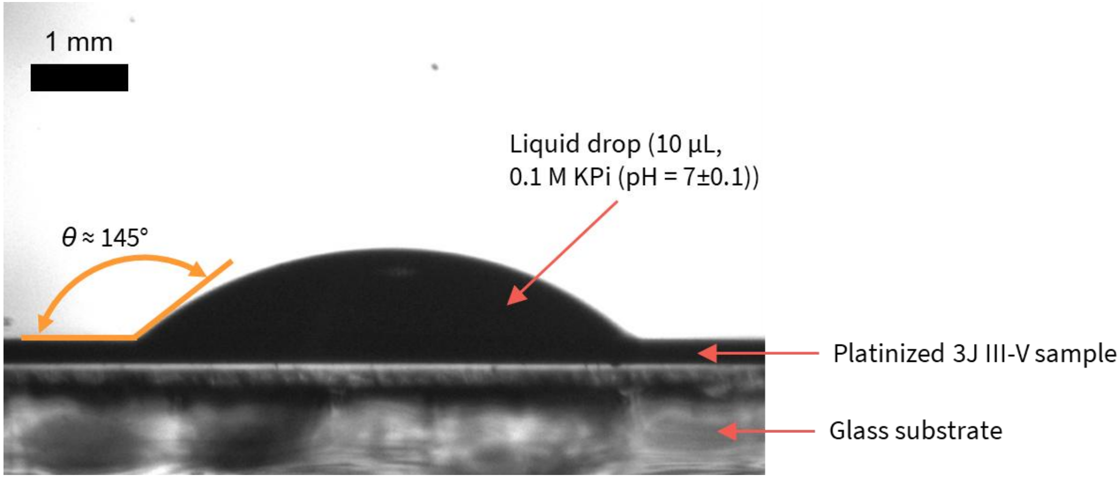

Figure S20. Contact angle measurement of a liquid droplet on the platinized 3J III-V photoelectrode. 10 μL of the electrolyte (0.1 M KP_i, pH = 7±0.1) was carefully dropped onto the sample, and the images were captured using a high-speed camera. The measured contact angle on the gaseous phase side was approximately 145°. The measurements were conducted at room temperature (~25 °C).

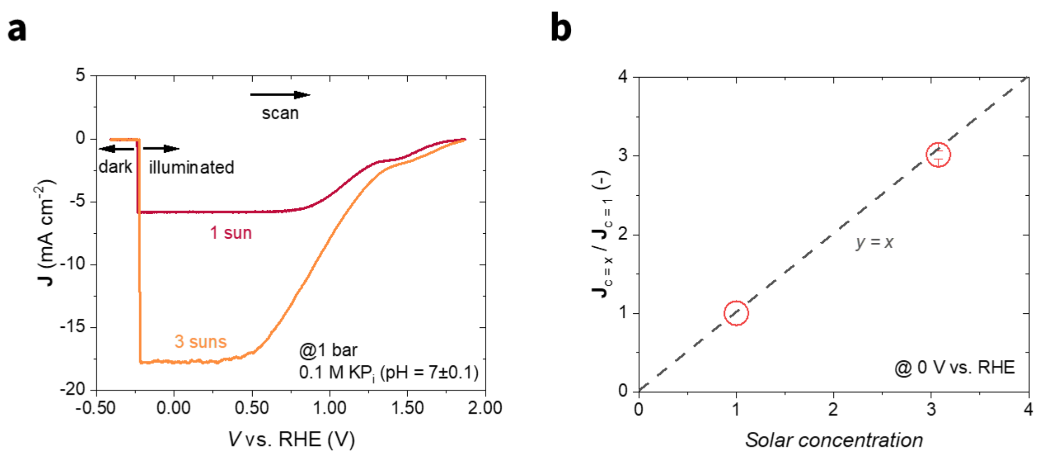

Figure S21. (a) Photocurrent vs. voltage curves of the platinized 3J III-V photoelectrode under different solar concentrations in 0.1 M KP_i (pH = 7±0.1). (b) Normalized photocurrent measured at 0 V vs. RHE. A Fresnel lens with a 600 mm focal length was used for this measurement, as shown in the solar irradiance data in Fig. S3. Error bars in panel (b) represent the standard deviations of the normalized photocurrent obtained from three different samples. The measurements were conducted at room temperature (~25 °C). No iR corrections was done for the reported voltammograms.

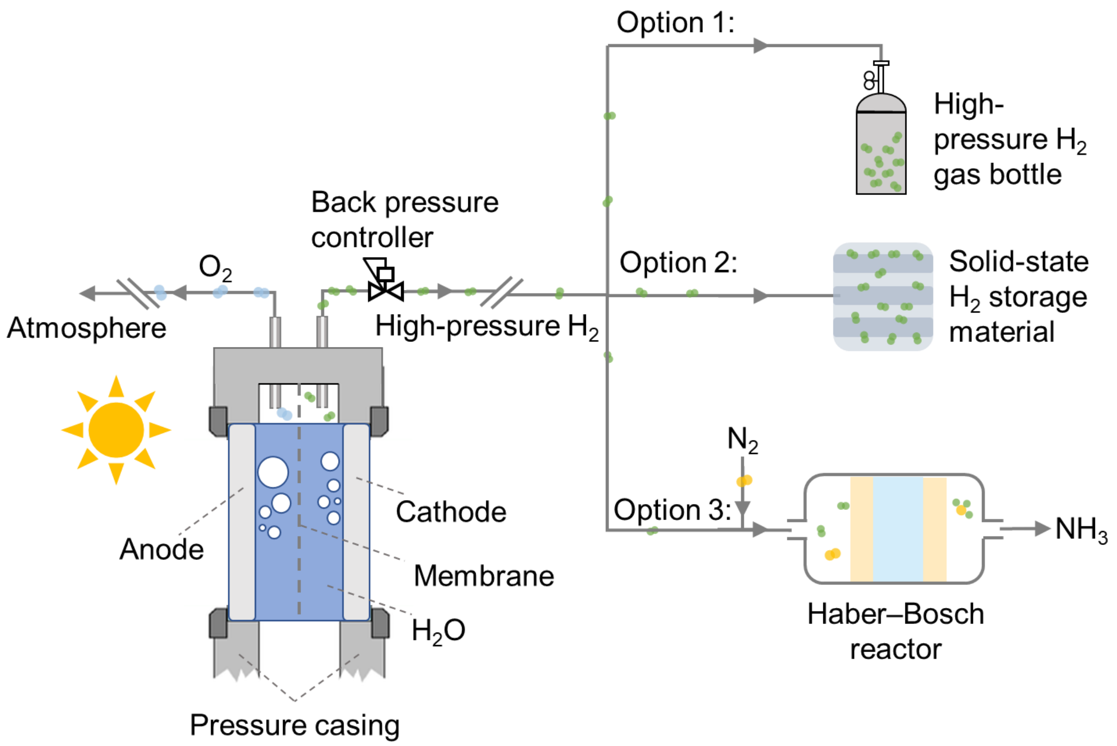

Figure S22. Schematic illustrations of three possible strategies for high-pressure H_2 storage/utilization. Option 1: the high-pressure H_2 is stored in a gas cylinder; Option 2: the high-pressure H_2 is absorbed by the solid-state H_2 storage materials, such as MgNi, ZrCr, and $LaNi_5$-based metal hydrides; Option 3: the high-pressure H_2 is directly fed into high-pressure hydrogen applications such as a Haber–Bosch reactor. Note that the oxygen is produced and released to the environment at nearly atmospheric pressure. Such a differential- pressure operation can be enabled by using a proton-exchange-membrane (e.g., Nafion[@] 117). Further note that the modules such as gas-liquid separator, H_2 gas purification are not included in this schematic illustration.

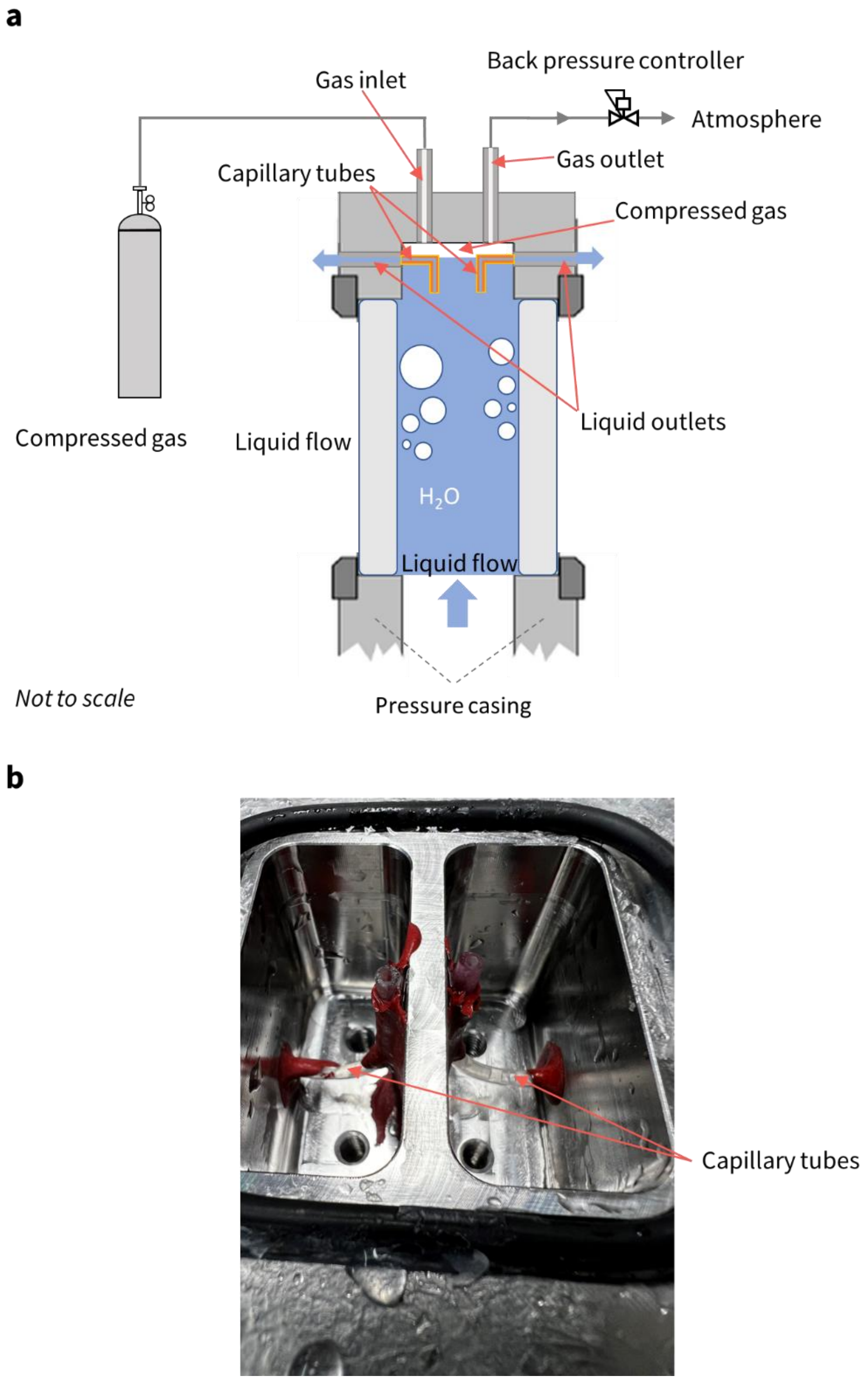

Figure S23. (a) Schematic illustration of the liquid outlets, and the gas inlets and outlets in our HPFC. (b) Digital photograph of a close-up view of the capillary tube. These capillary tubes (inner diameter of ~1.5 mm) were inserted underneath the electrolyte level to minimize the undesired cavitation during experiments.

References

1 Gennes, P.-G., Brochard-Wyart, F. & Quéré, D. *Capillarity and wetting phenomena: drops, bubbles, pearls, waves*. (Springer, 2004).

2 Butt, H.-J., Graf, K. & Kappl, M. *Physics and chemistry of interfaces*. (John Wiley & Sons, 2023).

3 Jain, S. & Qiao, L. Molecular dynamics simulations of the surface tension of oxygen-supersaturated water. *AIP Advances* **7** (2017).

4 Holmes-Gentle, I., Tembhurne, S., Suter, C. & Haussener, S. Kilowatt-scale solar hydrogen production system using a concentrated integrated photoelectrochemical device. *Nature Energy*, 1-11 (2023).

5 Zhang, L. *et al.* Significantly enhanced photocurrent for water oxidation in monolithic Mo: BiVO 4/SnO 2/Si by thermally increasing the minority carrier diffusion length. *Energy & Environmental Science* **9**, 2044-2052 (2016).

6 Bedoya-Lora, F. E., Holmes-Gentle, I., Feurstein, P. & Haussener, S. Effect of Operating Conditions on the Degradation of BiVO4 Photoanodes. *Advanced Functional Materials*, 2505102

7 Tembhurne, S., Nandjou, F. & Haussener, S. A thermally synergistic photo-electrochemical hydrogen generator operating under concentrated solar irradiation. *Nature Energy* **4**, 399-407 (2019).

8 Holmes-Gentle, I., Bedoya-Lora, F. E., Aimone, L. & Haussener, S. Photoelectrochemical behaviour of photoanodes under high photon fluxes. *Journal of Materials Chemistry A* **11**, 23895-23908 (2023).

9 Moon, C., Seger, B., Vesborg, P. C. K., Hansen, O. & Chorkendorff, I. Wireless photoelectrochemical water splitting using triple-junction solar cell protected by TiO2. *Cell Reports Physical Science* **1** (2020).
