# Supplementary material for: Photoelectrochemical water splitting cells at elevated pressure using BiVO4 and platinized III-V semiconductor photoelectrodes
Source: Nat Commun. 2025 Dec 13;16:11139. doi: 10.1038/s41467-025-67294-3 (PMC12706061; doi:10.1038/s41467-025-67294-3)
Supplement: Supplementary file 2 — Description of Additional Supplementary Files [file 41467_2025_67294_MOESM2_ESM.pdf]

Description of Additional Supplementary Files

Supplementary Video 1

Gas bubble evolution during the linear sweeping voltammetry (LSV) measurement of the back-illuminated, $BiVO_4$-based PEC cell at 1 bar. The electrolyte is a 0.1 M KP_i (pH =7) buffer solution with 0.5 M Na_2SO_3 added as a hole scavenger. The working electrode (WE) is $BiVO_4$ photoanode (visible on the left-handed side), a platinum mesh is used as the counter electrode (CE, visible on the right-handed side), with Ag/AgCl severed as the reference electrode (RE, not shown in the video). The video presents a side-view of gas bubble evolution from both the WE and CE. The electrolyte was circulated at a flow rate of ~4.6 mL s^{-1}. The electrolyte was purged using O_2 before experiment, and the operating pressure was maintained at 1 bar. The solar concentration was ~10 suns.

Supplementary Video 2

Gas bubble evolution during the LSV measurement of the back-illuminated $BiVO_4$-based PEC cell in a 0.1 M KP_i (pH =7) buffer solution at 1 bar. The working electrode (WE) is $BiVO_4$ photoanode (visible on the left-handed side), a platinum mesh is used as the counter electrode (CE, visible on the right-handed side), with Ag/AgCl severed as the reference electrode (RE, not shown in the video). The video presents a side-view of gas bubble evolution from both the WE and CE. The electrolyte was circulated at a flow rate of ~4.6 mL s^{-1}. Compressed O_2 gas was used to purge the electrolyte before experiments. The solar concentration was ~10 suns.

Supplementary Video 3

Gas bubble evolution during the LSV measurement of the back-illuminated $BiVO_4$-based PEC cell in a 0.1 M KP_i (pH =7) buffer solution at 3 bar. The working electrode (WE) is $BiVO_4$ photoanode (visible on the left-handed side), a platinum mesh is used as the counter electrode (CE, visible on the right-handed side), with Ag/AgCl served as the reference electrode (RE, not shown in the video). The video presents a side-view of gas bubble evolution from both the WE and CE. The electrolyte was circulated at a flow rate of ~4.6 mL s^{-1}. Compressed O_2 gas was used to purge and pressurize the system. The solar concentration was ~10 suns.

Supplementary Video 4

Gas bubble evolution during the LSV measurement of the back-illuminated $BiVO_4$-based PEC cell in a 0.1 M KP_i (pH =7) buffer solution at 5 bar. The working electrode (WE) is $BiVO_4$ photoanode (visible on the left-handed side), a platinum mesh is used as the counter electrode (CE, visible on the right-handed side), with Ag/AgCl served as the reference electrode (RE, not shown in the video). The video presents a side-view of

gas bubble evolution from both the WE and CE. The electrolyte was circulated at a flow rate of ~4.6 mL s^{-1}. Compressed O_2 gas was used to purge and pressurize the system. The solar concentration was ~10 suns.

Supplementary Video 5

Gas bubble evolution during the LSV measurement of the front-illuminated, platinized 3J III-V based PEC cell a 0.1 M KP_i (pH =7) buffer solution at 1 bar. The working electrode (WE) is the platinized 3J III-V photocathode (visible on the left-handed side, tilted at ~45° from the horizontal to reduce the bubble plume density for better bubble imaging), an IrO_x/TaO_x/Ti mesh is used as the counter electrode (CE, not visible in the video), with Ag/AgCl severed as the reference electrode (RE, not shown in the video). The video presents a side-view of gas bubble evolution from the WE. The electrolyte was circulated at a flow rate of ~4.6 mL s^{-1}. Compressed N_2 gas was used to purge the electrolyte before the experiments. The solar concentration was 1 sun.

Supplementary Video 6

Gas bubble evolution during the LSV measurement of the front-illuminated, platinized 3J III-V based PEC cell a 0.1 M KP_i (pH =7) buffer solution at 3 bar. The working electrode (WE) is the platinized 3J III-V photocathode (visible on the left-handed side, tilted at ~45° from the horizontal to reduce the bubble plume density for better bubble imaging), an IrO_x/TaO_x/Ti mesh is used as the counter electrode (CE, not visible in the video), with Ag/AgCl severed as the reference electrode (RE, not shown in the video). The video presents a side-view of gas bubble evolution from the WE. The electrolyte was circulated at a flow rate of ~4.6 mL s^{-1}. Compressed N_2 gas was used to purge and pressurize the system. The solar concentration was 1 sun.

Supplementary Video 7

Gas bubble evolution during the LSV measurement of the front-illuminated, platinized 3J III-V based PEC cell a 0.1 M KP_i (pH =7) buffer solution at 5 bar. The working electrode (WE) is the platinized 3J III-V photocathode (visible on the left-handed side, tilted at ~45° from the horizontal to reduce the bubble plume density for better bubble imaging), an IrO_x/TaO_x/Ti mesh is used as the counter electrode (CE, not visible in the video), with Ag/AgCl severed as the reference electrode (RE, not shown in the video). The video presents a side-view of gas bubble evolution from the WE. The electrolyte was circulated at a flow rate of ~4.6 mL s^{-1}. Compressed N_2 gas was used to purge and pressurize the system. The solar concentration was 1 sun.

Supplementary Video 8

Gas bubble evolution during the LSV measurement of the front-illuminated, platinized 3J III-V based PEC cell a 0.1 M KP_i (pH =7) buffer solution at 8 bar. The working

electrode (WE) is the platinized 3J III-V photocathode (visible on the left-handed side, tilted at ~45° from the horizontal to reduce the bubble plume density for better bubble imaging), an $IrO_x/TaO_x/Ti$ mesh is used as the counter electrode (CE, not visible in the video), with Ag/AgCl severed as the reference electrode (RE, not shown in the video). The video presents a side-view of gas bubble evolution from the WE. The electrolyte was circulated at a flow rate of ~4.6 mL s^{-1}. Compressed N_2 gas was used to purge and pressurize the system. The solar concentration was 1 sun.

Supplementary Video 9

Gas bubble evolution during the LSV measurement of the front-illuminated, platinized 3J III-V based PEC cell at 8 bar. The electrolyte is a 0.1 M KP_i (pH =7) buffer solution containing 0.5 M $Na_2S_2O_8$ as electron scavenger. The working electrode (WE) is platinized 3J III-V photocathode (visible on the left-handed side, tilted at ~45° from the horizontal to reduce the bubble plume density for better bubble imaging), an $IrO_x/TaO_x/Ti$ mesh is used as the counter electrode (CE, not visible in the video), with Ag/AgCl severed as the reference electrode (RE, not shown in the video). The video presents a side-view of gas bubble evolution from the WE. The electrolyte was circulated at a flow rate of ~4.6 mL s^{-1}. Compressed N_2 gas was used to purge and pressurize the system. The solar concentration was 1 sun.
